# Stent Omission after Ureteroscopy and Lithotripsy (SOUL) in the Michigan Urological Surgery Improvement Collaborative (MUSIC): study protocol for a pragmatic prospective combined randomized and observational clinical trial

**DOI:** 10.1186/s13063-024-08587-8

**Published:** 2024-12-04

**Authors:** Russell E. N. Becker, Stephanie Daignault-Newton, Elaina Shoemaker, Dennis Sitek, Jennifer M. Thelus, Sabrina Clark, Adam Martin-Schwarze, Catherine Spino, Noelle E. Carlozzi, William J. Meurer, Anne E. Sales, Casey A. Dauw, Khurshid R. Ghani

**Affiliations:** 1 Department of Urology, University of Michigan, Ann Arbor, MI USA; 2 Department of Biostatistics, School of Public Health, University of Michigan, Ann Arbor, MI USA; 3 Center for Clinical Outcomes Development and Application, University of Michigan, Ann Arbor, MI USA; 4 Department of Emergency Medicine, University of Michigan, Ann Arbor, MI USA; 5 Department of Neurology, University of Michigan, Ann Arbor, MI USA; 6 Sinclair School of Nursing, University of Missouri, Columbia, MO USA

**Keywords:** Kidney stones, Nephrolithiasis, Lithotripsy, Ureteroscopy, Ureteral stents, Surgical decision-making, Patient-reported outcomes, Stent-related symptoms, Practice patterns

## Abstract

**Background:**

Ureteral stents are placed by urologists to ensure ureteral patency in the postoperative period following ureteroscopy to treat kidney stones, with the goal to reduce complications. However, ureteral stents themselves cause pain and urinary symptoms in many patients that can lead to morbidity. Professional society guidelines support stent omission after uncomplicated ureteroscopy, which represents most cases. Despite this, ureteral stents are utilized in more than 80% of all ureteroscopy procedures. One reason for guideline discordance is the low level of evidence supporting stent omission recommendations. Studies are inconclusive on whether stents increase pain and complications. A recent Cochrane review concluded higher quality and large trials are needed to inform decision-making. Furthermore, there is a lack of studies evaluating health-related quality of life (HRQOL), patient-reported outcomes (PROs), and unplanned healthcare utilization. Another factor is that prospective clinical trials are hindered by patient reluctance to be randomized to either stent placement or omission. The outcomes of patients who decline randomization have been ignored in trials, limiting the generalizability of the evidence.

**Methods:**

Through collaboration with patient partners, we developed a pragmatic multi-center combined randomized and observational cohort study in a quality improvement collaborative. Patients will be prospectively enrolled into a randomized cohort in which assignment to ureteral stent omission (vs. placement) is determined in the operating room using a web-based randomization platform. Patients who decline randomization are invited to take part in an observational (real-world) cohort in which the determination of stent use is at the discretion of the urologist. Patients in both cohorts will complete preoperative and postoperative assessments of PROs including pain, urinary symptoms, interference with usual activities, time taken off work or school, and treatment satisfaction. Unplanned healthcare utilization within 30 days postoperatively will be assessed by review of the electronic health record. Severe adverse events will be recorded. A subgroup of patients and urologists will also participate in qualitative semi-structured interviews focusing on knowledge, preferences, and practice patterns regarding ureteral stenting. Interview transcripts will be thematically analyzed.

**Discussion:**

This study is designed to evaluate the HRQOL and 30-day healthcare utilization of patients undergoing ureteral stent omission compared to stent placement following uncomplicated ureteroscopic treatment of upper urinary tract stones. Additionally, patient and urologist opinions and preferences related to ureteral stenting will be explored through qualitative interviews, with the aim of identifying key barriers and facilitators of practice change related to stenting practices.

**Trial registration:**

ClinicalTrials.gov, NCT05866081. Registered on 19 May 2023.

## Administrative information

Note: the numbers in curly brackets in this protocol refer to SPIRIT checklist item numbers. The order of the items has been modified to group similar items (see http://www.equator-network.org/reporting-guidelines/spirit-2013-statement-defining-standard-protocol-items-for-clinical-trials/).

**Table:** 

Title {1}	Stent Omission after Ureteroscopy and Lithotripsy (SOUL) in the Michigan Urological Surgery Improvement Collaborative (MUSIC): Study protocol for a pragmatic prospective combined randomized and observational clinical trial of ureteral stent omission after ureteroscopic stone treatment
Trial registration {2a and 2b}.	NCT05866081 [ClinicalTrials.gov; registered 19 May 2023]
Protocol version {3}	Version 1.0 of 1st June, 2023
Funding {4}	This study is funded by the Patient-Centered Outcomes Research Institute (PCORI) Award (CER-2021C2-22,856). The Michigan Urological Surgery Improvement Collaborative is funded by Blue Cross Blue Shield of Michigan.
Author details {5a}	RENB: Department of Urology, University of Michigan SDN: Department of Urology, University of Michigan ES: Department of Urology, University of Michigan DS: Patient Partner JMT: Department of Urology, University of Michigan SC: Department of Urology, University of Michigan CS: Department of Biostatistics, University of Michigan School of Public Health NEC: Center for Clinical Outcomes Development and Application, University of Michigan WM: Departments of Emergency Medicine and Neurology, University of Michigan AES: Sinclair School of Nursing, University of Missouri CAD: Department of Urology, University of Michigan KRG: Department of Urology, University of Michigan
Name and contact information for the trial sponsor {5b}	Investigator-initiated clinical trial; K.R. Ghani (Principal Investigator)kghani@med.umich.edu
Role of sponsor {5c}	The investigators were responsible for the trial design, drafting of this manuscript, and the decision to submit the trial protocol for publication. The study design, analysis, and interpretation of results presented in this publication are solely the responsibility of the authors and do not necessarily represent the views of the Patient-Centered Outcomes Research Institute® (PCORI®), its Board of Governors or Methodology Committee.

## Introduction

### Background and rationale {6a}

In the USA, one in ten adults will have a kidney stone in their lifetime [1]. This chronic disease is recurrent and characterized by severe pain [2]. For patients unable to pass a stone, surgery is the mainstay of treatment [3]. With over 750,000 performed a year, ureteroscopy and lithotripsy are the most common procedure to remove or break up stones [4, 5]. At the end of ureteroscopy, a urologist may insert a temporary ureteral stent, a plastic flexible tube that allows urine to drain from the kidney to the bladder, which is later removed in the office, or at home by patient self-removal, which itself can provoke anxiety for patients [6, 7].

While the decision to place a ureteral stent is often made with good intentions, it is often unnecessary. Current US practice guidelines recommend stent omission if ureteroscopy is uncomplicated (e.g., no ureteral injury, stricture, or anatomic abnormality), which represents 80–90% of all cases. Despite this, stents are placed in up to 80% of all patients [8–10]. This utilization of stents comes at a cost to patients. Stents can have a detrimental effect on patient health-related quality of life (HRQOL), i.e., the impact that a disease has on mental, physical, and social well-being [11]. Stents result in flank pain, blood in the urine, and urinary symptoms in 80% of patients [12]. Data shows that 12.7–16.9% of patients visit the emergency department after ureteroscopy because of post-operative symptoms [8, 13–15], and 4–5.8% of these patients are hospitalized [16–19]. Total costs arising from stent-related problems while the stent is in place have been calculated to be a median $455 per patient (range $113–11,948) [20], making this among the costliest urologic disease to treat (expenditures in the US exceed $10 billion per year) [21]. Many patients develop stone recurrence during their lifetime, leading to multiple procedures and suffering.

A major reason for guideline discordance is the low level of evidence supporting stent omission recommendations. Studies are inconclusive on whether stents increase pain and complications. A recent Cochrane review concluded higher quality and large trials are needed to inform decision-making in this high-impact area [22]. Importantly, there is a lack of studies evaluating HRQOL, patient-reported outcomes (PROs), and unplanned healthcare utilization. Another factor is that because of experience with ureteroscopy, patients may decline to participate in randomized clinical trials (RCTs) of ureteral stents. The outcomes of these patients have been ignored in trials, limiting the generalizability of the evidence. Combined randomized and observational studies can address this gap. Finally, surgeon and patient preferences on stenting are not characterized, leaving an opportunity to inform strategies targeting practice change.

There are multiple limitations in studies that have assessed this topic. A Cochrane review of the comparative effectiveness of stent omission vs. placement after uncomplicated ureteroscopy (16 RCTs consisting of 1970 participants) found a trend for stenting to reduce the number of unplanned visits [22]. However, the included studies were limited by low confidence of evidence, performance bias, inconsistency, and imprecision, prohibiting clear interpretation of these results. To date, all RCTs assessing omission vs. placement have been performed at academic centers with small sample sizes [22].

The Michigan Urological Surgery Improvement Collaborative (MUSIC), established in 2011, is a physician-led quality improvement collaborative comprised of 44 urology practices and 260 urologists (90% of practicing urologists) across the State of Michigan and 3 centers outside Michigan [23]. Support for MUSIC is provided by Blue Cross Blue Shield of Michigan. MUSIC is a community that partners to improve patients’ lives by inspiring high-quality care through data-driven best practices, education, and innovation. The collaborative is designed to evaluate and improve the quality and cost efficiency of urologic care. By implementing changes in clinical behavior, MUSIC achieves more efficient utilization of healthcare resources, improves care delivery, and enhances the quality, value, and outcomes of treatment provided to urologic patients.

The objective of the Stent Omission after Ureteroscopy and Lithotripsy (SOUL) study is to compare patient outcomes from stent omission versus stent placement after uncomplicated ureteroscopy, as previously defined using RAND Appropriateness Methodology by MUSIC [24]. We will assess PROs (HRQOL, symptoms, treatment satisfaction) healthcare utilization, and factors associated with decision-making for stenting in patients undergoing ureteroscopy in MUSIC, comprised of diverse urology practices in Michigan, as well as MUSIC-affiliated practices outside Michigan. Our proposal is driven by patient partners and supported by a statewide clinical registry and PRO system, allowing unprecedented efficiency to conduct clinical trials in a real-world setting. The combined randomized and observational trial design assesses outcomes and preferences in all patients. We hypothesize that stent omission after uncomplicated ureteroscopy and lithotripsy will be associated with improvements in PROs (HRQOL, symptoms, treatment satisfaction) and 30-day healthcare utilization.

### Objectives {7}

#### Primary objectives

- To compare patient-reported pain interference 1 week after ureteroscopy between ureteral stent omission versus stent placement treatment arms
- To compare 30-day unplanned stone-related healthcare utilization between ureteral stent omission versus stent placement treatment arms

#### Secondary objectives

- To compare 30-day unplanned stone-related healthcare utilization at each level of the composite score between ureteral stent omission versus stent placement treatment arms
- To assess pain and health-related quality of life (HRQOL) in patients following ureteroscopy between ureteral stent omission versus stent placement treatment arms
- To assess urinary symptoms in patients following ureteroscopy between ureteral stent omission versus stent placement treatment arms
- To compare treatment satisfaction between ureteral stent omission versus stent placement treatment arms
- To compare time off work for patients and caregivers between ureteral stent omission versus stent placement treatment arms

#### Exploratory objectives

- To compare patient-reported daily pain scores between ureteral stent omission versus stent placement treatment arms
- To compare patient-reported interference with the performance of usual work between ureteral stent omission versus stent placement treatment arms
- To compare pain medication usage between ureteral stent omission versus stent placement treatment arms
- To compare the incidence of abnormal postoperative imaging findings after ureteroscopy between ureteral stent omission versus stent placement treatment arms
- To compare stone treatment success (stone-free rates) between ureteral stent omission versus stent placement treatment arms
- To compare patient-reported pain, HRQOL, urinary symptoms, unplanned healthcare utilization, and treatment satisfaction between patients undergoing uncomplicated ureteroscopy in the randomized controlled trial and the observational cohort study
- To evaluate patients’ and surgeons’ prior knowledge, opinions, and preferences regarding stent omission versus stent placement following ureteroscopy for stone disease

### Trial design {8}

**Fig. 1:**
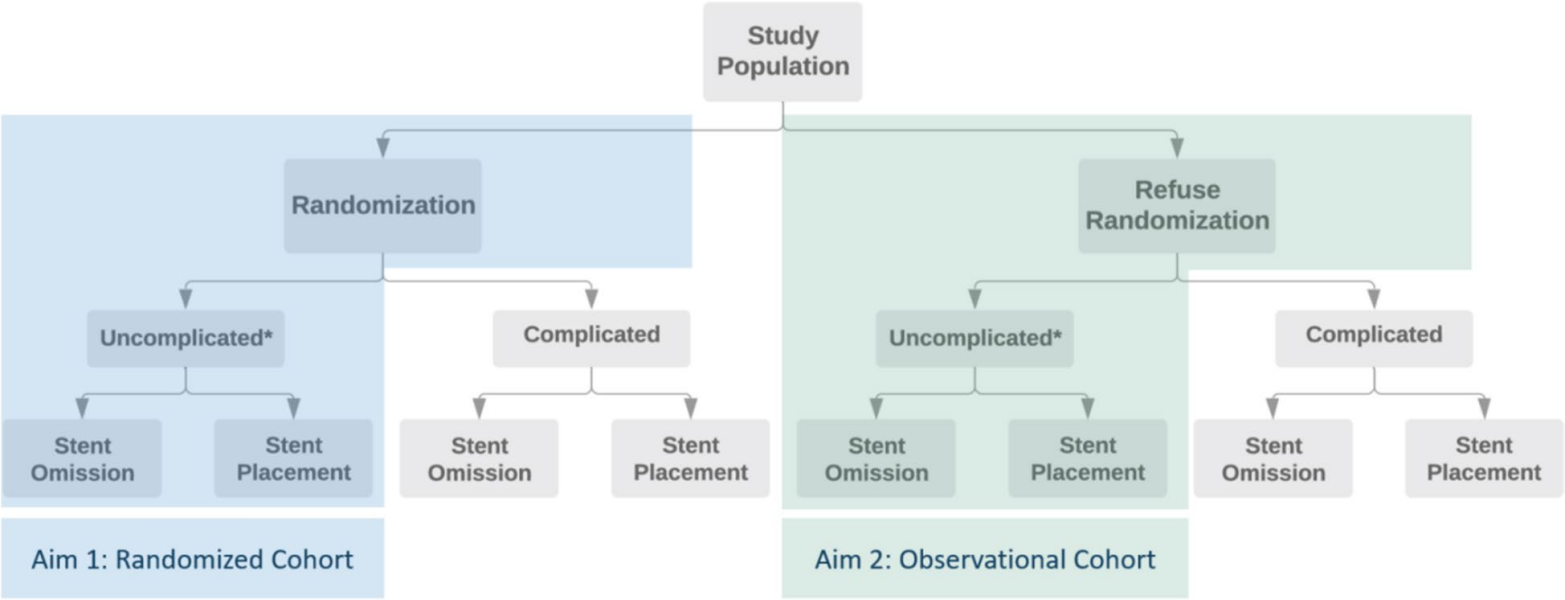
Overall design of the Stent Omission after Ureteroscopy and Lithotripsy (SOUL) study, including both cohorts

SOUL is a pragmatic multicenter comparative effectiveness prospective trial with randomized and observational cohorts assessing PROs and unplanned healthcare utilization following ureteroscopic treatment of patients with renal and ureteral stones, undergoing either stent omission versus placement. Qualitative interviews with selected patient and urologist participants in the trial, as well as surveys to all patients, will also be conducted to elicit preferences and opinions regarding ureteral stenting practices. A graphical representation of the overall study design is shown in Fig. 1.

## Methods: participants, interventions, and outcomes

### Study setting {9}

Participants will be enrolled at multiple urology practices that are part of MUSIC, consisting of various sizes and practice settings (academic centers, community hospitals, private provider groups) throughout the USA. The current list of participating sites can be found on ClinicalTrials.gov. We anticipate 14 centers throughout the country.

### Eligibility criteria {10}

Primary inclusion criteria: patients must meet all of the following criteria to be eligible for participation in the study:

- Age ≥ 18 years
- Undergoing unilateral ureteroscopy and lithotripsy for stone disease (participants may have contralateral stones, as long as these are asymptomatic and not being treated concurrently)
- Largest stone ≤ 10 mm in size as measured on abdominal x-ray, ultrasound, or CT scan
- Access to means of communication with the study team (email, text messaging, and/or telephone)
- Adequate independent cognitive function and English language proficiency to complete study surveys
- Written informed consent

Primary exclusion criteria: If the patient meets any of the following criteria preoperatively, they will not be eligible for participation in the study:

- Planned bilateral ureteroscopy
- Indwelling ureteral stent or percutaneous nephrostomy tube preoperatively in either kidney
- Anatomic abnormalities of the ipsilateral upper urinary tract (e.g., horseshoe kidney, crossed fused ectopia, pelvic kidney, urinary diversion)
- Anatomic or functional solitary kidney
- Planned secondary or staged ureteroscopy
- Planned use of a ureteral access sheath during ureteroscopy
- Pregnancy
- Patients who use opiate medication daily for > 3 months to manage a painful condition

Second-stage eligibility criteria: if any of the following criteria are met intraoperatively, the patient will not be eligible for participation in the study:

- Ureteral perforation
- Unanticipated anatomic abnormality (e.g., ureteral stricture or ureteropelvic junction obstruction)
- Greater than expected bleeding
- Ureteral dilation performed (> 12 French)
- Ureteral access sheath utilized
- Failed ureteroscopy (unable to treat stone, requiring stent, or nephrostomy)
- No lithotripsy performed (e.g., no stone found)
- Intraoperative decision to perform incomplete lithotripsy (e.g., and return later for staged procedure)
- Unable to complete case due to medical or anesthetic event
- Other (will need to be discussed with coordinating center PI within 7 days)

### Who will take informed consent? {26a}

Patients scheduled to undergo unilateral ureteroscopy for treatment of upper urinary tract stones at participating sites will be screened for eligibility by trained study coordinators based on the aforementioned primary inclusion criteria and primary exclusion criteria. Names and identifiers of potential participants will be securely sent to the treating urologist for confirmation. Upon confirmation of eligibility by the treating urologist, patients will be contacted by trained study coordinators to discuss the details of the study and sign informed consent for participation. A patient-facing video explaining the clinical trial, made with commentary from the investigators, patient partners, and trial coordinating team members will be created, so it can be used at all sites as part of trial counseling.

Study materials and consent materials will be provided to the patient via an E-consenting platform. Paper consent forms using handwritten signatures may also be used in lieu of E-consenting. The participant will sign the informed consent document prior to any study-specific procedures being performed. A copy of the informed consent document will be given to the participants for their records, and an electronic copy will be appended to their local electronic medical record.

### Additional consent provisions for collection and use of participant data and biological specimens {26b}

Not applicable. No biological specimens will be collected.

## Interventions

### Explanation for the choice of comparators {6b}

The SOUL study is a pragmatic comparison between two existing management strategies that are both widely considered routine standard clinical care. All potential participants are planning to undergo ureteroscopy with a urologist trained in ureteroscopy in accordance with American Urological Association (AUA) guidelines. The intervention in this trial is stent omission. Patients will either continue to receive a ureteral stent with the surgeon’s choice of stent, or undergo stent omission, both of which are widely accepted as standard clinical care. In the observational cohort, assignment to stent omission will be at the discretion of the operating urologist per routine clinical practice, which may or may not include patient preferences. In the randomization cohort, assignment to stent omission versus stent placement will be by 1:1 randomization. When a stent is placed, it may or may not be left on a string (tether), at the operating urologist’s discretion. The duration of stenting, and how it is removed, is left to the discretion of the urologist.

### Intervention description {11a}

The only study-specific procedures representing anything more than routine clinical care are patient surveys. All participants in both the randomization and observational cohorts will receive PRO questionnaires via e-mail or alternative means of communication at several prespecified time points: pre-operatively, at 7–10 days after surgery, and at 4–6 weeks after surgery. If the patient is unable to complete the study questionnaires electronically, the patient may complete questionnaires over the telephone with assistance from a SOUL study team member or paper questionnaires upon request. Patients will also receive automated questionnaires (daily Ecological Momentary Assessments (EMA)) via text message, daily for 10 days after surgery and on day 30 after surgery. PRO and EMA responses will be recorded and secured.

We will also conduct semi-structured interviews with an initial subgroup of 20–30 study participants, striving for evenly balanced representation between the randomized and observational cohorts, to elicit prior knowledge, desires, preferences, and experiences regarding stent placement and stent omission. We will endeavor to keep patient interviews to less than 45 min. They will be conducted by phone or web-based conferencing service and recorded with the permission of the participant. Full transcripts will be made and anonymized for analysis, and the original recordings permanently deleted. Based upon the findings from these interviews, a survey will be developed and distributed to all participants in the study.

Similarly, we will conduct semi-structured interviews with urologists participating in the trial to evaluate perceptions, experience, preferences, and practice patterns regarding stent omission versus stent placement following uncomplicated ureteroscopy. We will also inquire about important factors and determinants in the urologist’s decisions to omit or place a ureteral stent. We will endeavor to interview all urologists participating in the trial or until we reach thematic saturation. We will strive to include representation from urologists in a variety of practice settings and include urologists both within and outside the state of Michigan. We will endeavor to keep urologist interviews to less than 30 min. They will be conducted by phone or web-based conferencing service and recorded with the permission of the participating urologist. Full transcripts will be made and anonymized for analysis, and the original recordings permanently deleted.

**Fig. 2:**
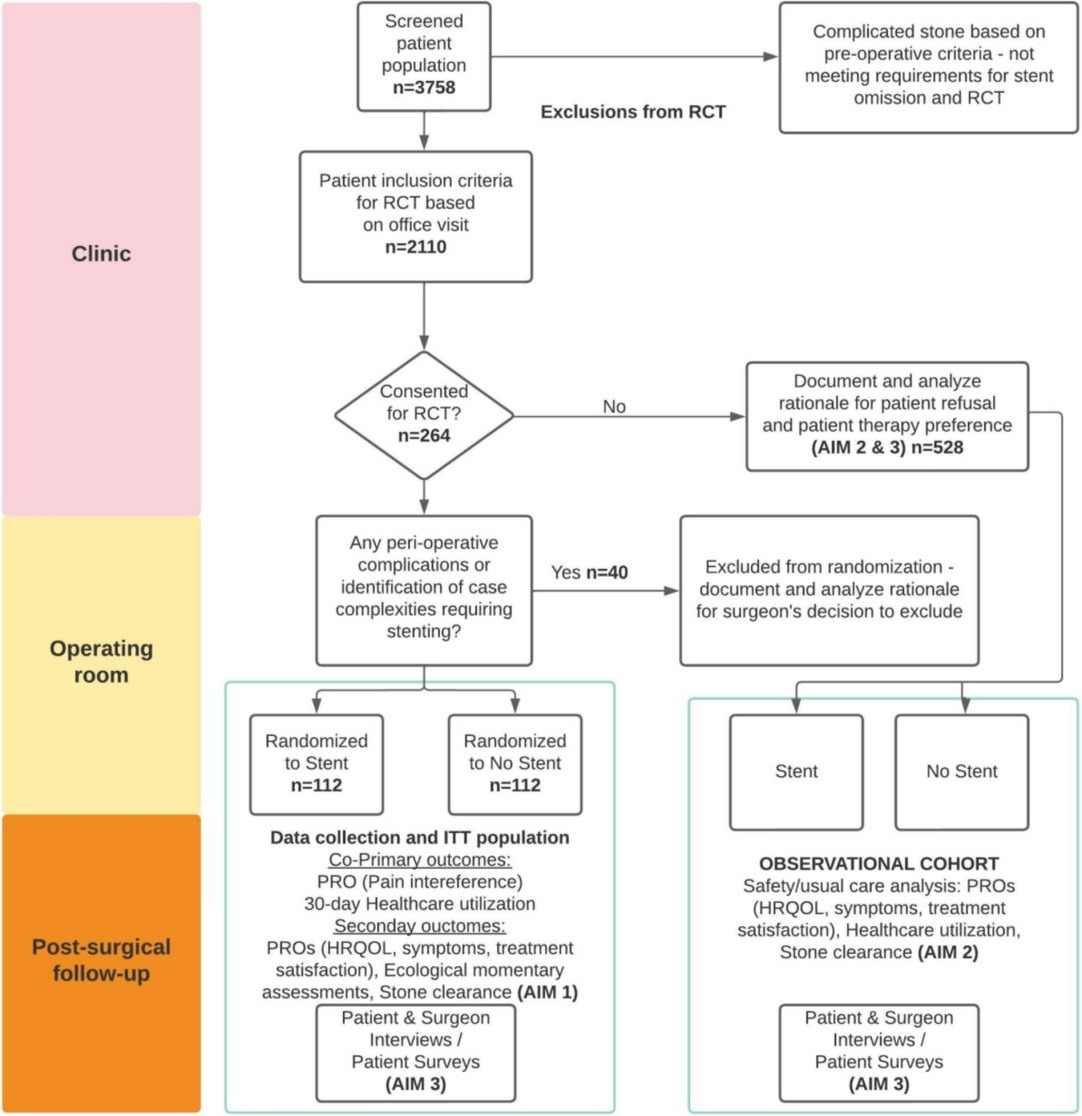
CONSORT flow diagram showing study design with intersection of aims

A CONSORT diagram of the detailed study design and workflow is provided in Fig. 2.

### Criteria for discontinuing or modifying allocated interventions {11b}

Stent omission (versus placement) is performed at a single time point intraoperatively and is not subject to discontinuation. However, a subject’s participation in patient surveys (PRO and EMA) may be discontinued at an investigator’s discretion for the following reasons:

- Significant study non-compliance or inability to meaningfully complete study surveys
- If the participant meets an exclusion criterion (either newly developed or not previously recognized) that precludes further study participation
- If continued participation in the study would not be in the best interest of the participant

Participants are also free to withdraw from participation in the study at any time upon request.

### Strategies to improve adherence to interventions {11c}

Adherence to the intervention (stent omission versus stent placement) occurs in the operating room at a single time point near the conclusion of surgery and is dependent upon proper understanding of the trial and workflow by the surgeon and staff. Therefore, each site’s participating urologists and staff receive an extensive site initiation visit and orientation with coordinating center staff and investigative team members prior to beginning their participation in the trial, and multiple lines of open communication including an Acute Patient Hotline are maintained to facilitate quick answers to any questions or issues that may arise. A dedicated public web page (https://www.stentornot.com/) has also been created, containing information on workflows, inclusion and exclusion criteria, study documents, consent forms, and survey instruments, to facilitate on-demand refresher training and allow rapid accrual into the study, especially for patients presenting acutely through the emergency department with minimal lead time to coordinate enrollment. The web page also hosts patient educational materials and a patient-facing video to help inform and educate patients about the study.

### Relevant concomitant care permitted or prohibited during the trial {11d}

The pragmatic nature of the study enables continued standard postoperative care as directed by the treating urologist. There are no study-specific restrictions on allowable concomitant care or interventions.

### Provisions for post-trial care {30}

The trial does not specify any provisions or restrictions for post-trial care. Unplanned healthcare encounters and unplanned secondary procedures within the postoperative data collection period will be recorded and analyzed as part of the unplanned healthcare utilization outcome metric.

### Outcomes {12}

Outcomes will be assessed and analyzed similarly in both the randomized and observational cohorts within the study. SOUL has two co-primary outcomes:

- Patient-Reported Outcomes Measurement Information System (PROMIS) Pain Interference (Short Form 6b) T-score, change at postoperative days 7–10 compared to preoperative score
- Unplanned stone treatment-related healthcare utilization within 30 days of ureteroscopy; a hierarchical composite comprised of:
  ◦ Intensive care unit (ICU) care during hospitalization
  ◦ Unplanned hospitalization
  ◦ Unplanned additional procedure related to ureteroscopy: operating room or interventional radiology procedure
  ◦ Emergency department visit
  ◦ Unplanned clinic visit and/or diagnostic testing (urine testing and/or imaging)
  ◦ Number of ambulatory patient–provider interactions: phone calls/EMR messages

Secondary outcomes include the following:

- ICU care within postoperative day 30
- Unplanned hospital admission within postoperative day 30
- Unplanned additional procedure within postoperative day 30
- Emergency department visit within postoperative day 30
- Unplanned ambulatory urology office visit and/or diagnostic testing (urine testing and/or imaging) within postoperative day 30
- Number of ambulatory patient-provider interactions: phone calls/EMR messages within postoperative day 30
- PROMIS Pain Interference (Short Form 6b) T-scores change at postoperative 4–6 weeks compared to preoperative score
- PROMIS Pain Intensity (Short Form 3a) T-scores change at postoperative days 7–10 and 4–6 weeks compared to preoperative score
- Symptoms of Lower Urinary Tract Dysfunction Research Network (LURN) Symptom Index (SI-10) change at postoperative days 7–10 and 4–6 weeks compared to preoperative scores
- International Consultation on Incontinence Questionnaire Satisfaction (ICIQ-S) scores at postoperative days 7–10 and 4–6 weeks
- Number of days taken off work by patients and caregivers during the first 7 days after ureteroscopy

The following exploratory outcomes will also be assessed:

- Ecological momentary assessments (EMA) measured via daily text message on postoperative days 1–10
  ◦ Postoperative days 1–10 reported interference with the performance of usual work from PROMIS Ability to Participate in Social Roles & Activities
  ◦ Postoperative days 1–10 visual analog scale (VAS) pain scores
  ◦ Postoperative days 1–10 patient-reported pain medication utilization
- Abnormal postoperative imaging findings within 60 days postoperative
- Stone-free imaging outcomes within 60 days postoperative
- Patient interviews to evaluate prior knowledge and preferences about ureteral stent omission versus stent placement during ureteroscopy to treat kidney stones
- Surgeon interviews to evaluate preferences about ureteral stent omission versus stent placement during ureteroscopy to treat kidney stones
- Patient survey to evaluate prior knowledge and preferences about ureteral stent omission versus stent placement during ureteroscopy to treat kidney stones

### Participant timeline {13}

As part of standard care, all patients will undergo a routine preoperative evaluation. Table 1 shows a full schedule of study-related activities and assessments in accordance with SPIRIT guidelines.

**Table 1:** SOUL study participant schedule of activities

**Procedure**	**Prior to operation**	**Operative day (day 0)**	**Electronic survey assessments (days 1–10)**	**Electronic survey assessments (days 7–10)**	**Electronic survey assessments (days 22–42)**	**Electronic survey assessments (day 30)**
Informed consent	X					
Study education	X					
Demographics	X					
Medical History	X					
Randomization		X^a^				
Stent omission or placement		X				
EMA			X^1^			X
PRO survey	X^2^			X	X	
Patient survey on preferences/attitudes					X	

^1^Automated daily EMA invitations begin postoperative day 1 and continue through day 10 for all patients, with a final text sent on day 30 querying any patient-reported adverse events or unplanned healthcare utilization

^2^Preoperative PRO survey bundle should be completed within 30 days preoperatively

^a^For participants in the randomized cohort only

### Sample size {14}

The sample size was derived by the sample needed to power each co-primary endpoint for the RCT cohort. The larger sample size among the co-primary endpoints was used to estimate the final RCT cohort size. Each power calculation used a two-sided significance level of 2.5% to allow for two primary endpoints for a trial-level type I error of 5% as described below.

Postoperative days 7–10 PROMIS® Pain Interference change from pre-surgery is a co-primary endpoint; 101 participants per treatment arm will provide the RCT cohort with at least 90% power to detect a mean difference between stent placement vs. omission treatment groups of 5 points with an assumed standard deviation (SD) of 10 points, based on a two-sample *t* test (SAS 9.4), with a two-sided significance level of 2.5%. Previous work estimated that a clinically meaningful difference in PROMIS® Pain Interference is 2–6 points [25, 26]. A pilot study at our institution found an initial difference between stent omission and placement of 7.2 points with a SD of 13 for change and a SD of 10 at each time point. We expect our pilot may overestimate the treatment effect. Thus, we have targeted a difference of 5 points to represent a clinically significant target. A 10% loss to follow-up (failure to respond to the 7–10-day questionnaire) is assumed. Thus, 112 patients are needed per arm (total 224 patients). After consent, we estimate that 15% of patients who plan to be randomized will be unable to do so owing to intra-surgical factors that deem the procedure as complicated requiring 264 patients consented to the randomization cohort to attain 224 randomized patients.

Unplanned healthcare utilization within 30 days of ureteroscopy is the second co-primary endpoint.

With a modest probability of improvement in the reduction of healthcare utilization in ureteroscopy with stent omission, the impact in morbidity reduction and cost reduction would be great. There are no current estimates in the literature that help inform a minimum important difference for the win ratio estimate of healthcare utilization, our co-primary endpoint. So, we reasoned that a marginal probabilistic index, which is the effect size measure corresponding to the nonparametric Mann–Whitney *U* statistic that is used to test the hierarchical composite “win ratio” endpoint [27], of at least 67%, would be impactful as the study could conclude that a randomly chosen participant with stent omission has a two-thirds or greater probability of lower healthcare utilization than a randomly chosen participant treated with a stent. In the RCT with a two-sided 2.5% alpha and requiring 90% power to detect a treatment effect of 67%, 72 patients would be required per treatment arm. The sample size is based on the Finkelstein and Schoenfeld methods [28] for analysis of hierarchical composite endpoints, later called the win ratio by Pocock et al. based on the nonparametric Mann–Whitney *U* statistic [29]; the formula is provided in Yosef et al. [30].

The observational cohort sample size was derived from an assumption that 1/3 of patients approached and consented would be willing to be randomized. Thus, the observational cohort will consist of the estimated 2/3 (528) of patients who are approached and decline the RCT. With those assumptions, we find the following power for each primary endpoint.

Approximately 85% (449) of the observational cohort consented patients are expected to undergo an eligible ureteroscopy. From our prior data, approximately 70% (314) of these patients will receive a stent during ureteroscopy, and 135 (30%) will have stent omission. Assuming a 10% loss-to-follow-up (days 7–10 pain interference questionnaire not completed), this will result in 283 stent placement patients and 121 stent omission patients evaluable for the primary analysis of the observational cohort. Assuming a two-sided 2.5% type I error for each primary endpoint, 283 patients in the stent placement arm and 121 patients in the stent omission arm will provide 99.1% power to detect a clinically meaningful difference of 5 points between treatment arms for the change from pre-surgery in PROMIS® pain interference measure at days 7–10, assuming a standard deviation of 10 using a two-sample *t*-test. Sensitivity to the stented proportion and proportion agreeing to randomization (Table 2) demonstrates at least 84% power to detect a clinically significant difference in pain interference.

**Table 2:** Observational cohort power with sensitivity to our assumptions

Proportion agrees to randomization (total approached)	Proportion stented	Sample size total (stented:stent omission)	Power
33% (792)	70%	404 (121:283)	99.1%
	50%	404 (202:202)	99.7%
	25%	404 (101:303)	99.1%
50% (528)	70%	202 (141:61)	84.2%
	50%	202 (101:101)	90.0%
	25%	202 (51:151)	86.7%

The power estimates presented assume that each observation is independent. However, due to surgeon preference in the observational cohort, there may be a potential for clustering which could impact the power of the study. We expect that surgeons within a site may behave more similarly and the variability between sites will be the correlation that will be important. The intraclass correlation (ICC) across multiple studies and therapeutic areas has been shown to likely be small, with a range between 0.01 and 0.05 [31]. Using our planned sample sizes, we can estimate the sample size needed under a range of ICCs using a method described by Klar and Donner [32] with an estimate of the sample size inflation factor (IF) based upon the ICC and the average sample size per cluster. Table 3 provides the power from using the effective sample size and the assumed proportions for each arm (stent vs stent omission using standard power calculations for the two-arm mean difference using a *t*-test with a two-sided type I error of 0.025 with the assumptions used for Pain Interference described above.

**Table 3:** Power for pain interference co-primary endpoint in observational cohort with clustering effect (ICC), assuming 14 participating sites

Proportion agrees to randomization	Total approached	Final observational cohort sample size	Proportion stented	ICC group			
				**0**	**0.01**	**0.02**	**0.05**
33%	792	404	70%	99.1%	96.5%	92.3%	76.3%
			50%	99.7%	98.6%	96.1%	83.7%
			25%	99.1%	94.4%	89.0%	71.0%
50%	528	202	70%	84.2%	78.6%	73.4%	59.6%
			50%	90.0%	85.8%	81.2%	68.0%
			25%	86.7%	73.5%	68.0%	54.2%

For the co-primary endpoint of healthcare utilization in the observation cohort, if we assume that the stented proportion is 50%, with 202 participants per treatment group and a two-sided 2.5% type I error (alpha), we have 90% power to detect a treatment effect, marginal probabilistic index, of 60.1%. If the stented proportion is as low as 25% or as great as 75%, then we can infer that with 90% power, we will be able to detect a treatment effect between 60.1% and 64.3% as the sample size will be at least 101 per stent group.

### Recruitment {15}

To date, ten practices in Michigan who are members of MUSIC are taking part in the SOUL trial. In aggregate, these practices perform 2878 ureteroscopies annually, with an estimated 1960 meeting study eligibility criteria. MUSIC is also currently undergoing an expansion of membership to include practices outside Michigan and thus far four such practices have indicated their intention to participate in the SOUL study. These “Outdoor MUSIC” sites perform an estimated additional 1300 ureteroscopies annually, of which at least half are estimated to meet SOUL eligibility criteria.

## Assignment of interventions: allocation

### Sequence generation {16a}

Participants in the randomization cohort will be randomized intraoperatively using an online computer-based system (https://randomize.net/) to either stent omission or stent placement in a 1:1 ratio. This will take place after the completion of the lithotripsy portion of the procedure and verification of second-stage eligibility by the operating urologist. Blocked, stratified randomization based upon surgeon-classified stone location (renal only versus ureteral with or without renal), with random block sizes will be used to ensure group balance.

### Concealment mechanism {16b}

Surgeons will not perform intraoperative randomization on the web-based platform until they have completed the lithotripsy portion of the procedure and verified that the participant meets second-stage eligibility criteria. Thus, randomized allocation does not occur until the point of performing the assigned intervention.

### Implementation {16c}

The trial has a dedicated biostatistician who has programmed online randomization platform with the trial design and the online platform generated the allocation sequence to be implemented. The randomization of individual participants and assignment to intervention will occur intraoperatively, only after completion of the lithotripsy portion of the procedure and confirmation by the operating urologist that the participant meets second-stage eligibility.

## Assignment of interventions: blinding

### Who will be blinded {17a}

The operating urologist will need to perform the allocated treatment (stent omission or stent placement) and therefore cannot be blinded. Because the presence of a stent necessitates arrangements need to be made for future stent removal, patients will also be informed of the presence or absence of a stent immediately following their surgery and will not be blinded.

### Procedure for unblinding if needed {17b}

Not applicable. Neither urologists nor patients will be blinded.

## Data collection and management

### Plans for assessment and collection of outcomes {18a}

Participating MUSIC practices submit data to a web-based clinical registry maintained by the MUSIC coordinating center developed in conjunction with a vendor (ArborMetrix, Ann Arbor, Michigan). The registry includes approximately 150 unique variables with information on patient demographics; laboratory, imaging results; comorbid conditions; treatments; and patient outcomes including complications and mortality, among others. Data collection is guided by standard variable definitions and collaborative-wide operating procedures. In terms of quality assurance, members of the coordinating center conduct on-site audits to ensure the appropriate identification of cases and the integrity of data entered into the registry.

All participants in both the randomization and observational cohorts will receive PRO questionnaires via e-mail or alternative means of communication at several prespecified time points: pre-operatively, at 7–10 days after surgery, and at 4–6 weeks after surgery. If the patient is unable to complete the study questionnaires electronically, the patient may complete questionnaires over the telephone with assistance from a SOUL study team member or paper questionnaires upon request. Patients will also receive automated questionnaires (Daily Ecological Momentary Assessments, EMA) via text message, daily for 10 days after surgery and on day 30 after surgery. PRO and EMA responses will be recorded and secured.

### Plans to promote participant retention and complete follow-up {18b}

Participation in the SOUL study is short-term, and minimal loss to follow-up is expected. Follow-up is limited to only routine postoperative clinical care, and no study-specific visits or tests are required.

Patients will receive PRO surveys electronically via an automated system. If a patient does not log-in or fails to complete the preoperative PRO survey, they will receive an electronic automated reminder and will be contacted by the MUSIC Coordinating Center. If a patient does not log-in or fails to complete the 7-day survey, they will receive an electronic automated reminder and will be contacted by the MUSIC Coordinating Center to facilitate its completion by the end of post-operative day 10. The SOUL Study Team may also administer the PRO questionnaires via REDCap, phone, or paper if the participant is unable to complete surveys through the MUSIC Registry. Similar procedures will be used for the completion of the 4–6-week PRO surveys.

### Data management {19}

Study participant research data, which is for purposes of statistical analysis and scientific reporting, will be transmitted to and stored at ArborMetrix. The study data entry and study management systems used by clinical sites and by MUSIC research staff will be secured and password protected. Data are regularly audited by manual review for quality and fidelity as part of routine MUSIC operating procedures. At the end of the study, all study databases will be de-identified and archived at MUSIC.

### Confidentiality {27}

Participant confidentiality and privacy is strictly held in trust by the participating investigators, their staff, and the study coordinating center. Therefore, the study protocol, documentation, data, and all other information generated will be held in strict confidence.

All research activities will be conducted in as private a setting as possible.

The MUSIC coordinating center and duly authorized representatives, representatives of the Institutional Review Board (IRB), or regulatory agencies may inspect all documents and records required to be maintained by the investigator, including but not limited to medical records (office, clinic, or hospital) and pharmacy records for the participants in this study. The clinical study site will permit access to such records.

The study participant’s contact information will be securely stored at each clinical site for internal use during the study. At the end of the study, all records will continue to be kept in a secure location for as long a period as dictated by the reviewing IRB, Institutional policies, or PCORI requirements.

### Plans for collection, laboratory evaluation and storage of biological specimens for genetic or molecular analysis in this trial/future use {33}

Not applicable. No biological specimens will be collected.

## Statistical methods

### Statistical methods for primary and secondary outcomes {20a}

Co-primary outcome: postoperative days 7–10 PROMIS® Pain Interference change from pre-surgery.

#### Randomized cohort analysis

A linear (ANCOVA) model will be used on the intention to treat (ITT) population to compare the two treatments, with PROMIS Pain Interference *t*-score at postoperative days 7–10 as the dependent variable and treatment group, stratification factor (stone location [kidney or ureter]) and pre-surgery (baseline) Pain Interference *t*-score as the independent variables [33]. The adjusted parameter estimate for treatment group (stent omission vs. placement) will be reported with the associated 97.5% CI and *p*-value (based on a type 3 *F*-test) that allows testing the hypothesis that Pain Interference differs between the two treatment groups. Multiple imputation will be used to address missing data and will be described fully in the statistical analysis plan. Sensitivity analysis will be conducted with the same analysis in the pain-modified intent-to-treat (PmITT, see Sect. 20c) population to assess the robustness of the effect found in the primary analysis. Additional sensitivity analysis will be conducted if there is baseline imbalance.

#### Observational cohort analysis

The primary analysis for the observational study will be similar to the model described in the randomized cohort except additional independent patient and surgical baseline covariates (age, sex, body mass index (BMI), Charlson comorbidity index, pre-surgery depression t-score, pre-surgery anxiety *t*-score, narcotic use, stone location (kidney or ureter), and stone size) will be included in the model to adjust for potential confounders that are more likely to be unbalanced between the treatment groups owing to lack of randomization. Additionally, a multivariable model with random effects for physician or practice will be considered if there is further imbalance in these factors.

##### Co-primary outcome: unplanned healthcare utilization

Unplanned healthcare utilization related to surgery within 30 days of ureteroscopy, defined as a hierarchical composite rank score components in decreasing rank order as described in the protocol. The composite endpoint will be nonparametrically assessed by comparing global composite rank score outcomes [34] across pairs of participants and evaluating a win ratio using the Mann–Whitney *U* statistic. The win proportion is the probability that a stent omission participant experiences better outcomes when compared to a participant with stent, thus representing the effect size.

#### Randomized cohort analysis

The analysis of the hierarchical composite endpoint of healthcare utilization using the Win Ratio [29] in the RCT will use the ITT population and will use the unmatched approach [35, 36]. The composite healthcare utilization metric within 30 days will be an assigned score for each patient based upon the highest ureteroscopy-related utilization level that they participate in with levels defined in decreasing order: (6) Hospitalization and ICU care; (5) unplanned hospitalization; (4) unplanned additional operating room or interventional radiology procedure; (3) emergency department (ED) visit; (2) ambulatory encounter: clinic visit; and (1) number of ambulatory patient/provider interactions. Then the rank of each patient in the stent omission arm will be compared to each patient in the stent arm using the unplanned healthcare utilization ranking rules described above such that each pair will have a winner. Summary statistics for the hierarchical composite win ratio endpoint will be presented, including the number of total pairs, and number and proportion of pairs that favor stent omission for each component of the unplanned healthcare utilization outcome. The number of winners and losers for stent omission and corresponding win ratio will be reported with the 97.5% (bootstrapped-based) confidence interval and *p*-value based on the Chi-square statistic. In addition, the probability of a participant with stent omission doing better than a participant with a stent will be presented. It is not expected to have missing data for this endpoint but if there is then multiple imputation will be used. Sensitivity analysis will be conducted with the same analysis in the healthcare utilization modified intent-to-treat (HCUmITT, see Sect. 20c) population to assess the robustness of the effect found in the primary analysis.

#### Observational cohort analysis

The same general approach used in the RCT will be applied to the observational study with the healthcare utilization rank for each patient and the rules for comparing pairs of patients between arms. However, to address possible clustering among surgeons and sites while protecting unbiasedness of treatment effects and to preserve power, the analysis approach of Dong et al. [36] will be implemented. Specifically, this approach incorporates the notion of risk-matching by forming strata according to risk groups (as described by Pocock et al. [29]) without resulting in loss of information by losing matched pairs when there is an imbalance in sample size between groups. The stratified approach uses the unmatched method to calculate the win ratio statistics within stratum, thus providing an intermediate position between the matched pairs approach and the unmatched approach. Specifically, we will include site in the determination of risk strata. To form risk group strata, the outcome of healthcare utilization categorized as yes versus no will be modeled using a logistic model with fixed effects including baseline variables: site, stone location (kidney only vs ureter), stone size, and Charlson comorbidity score. A risk score for each patient will be obtained using the logistic model’s coefficients. Stratification will be performed based upon risk score. Risk score groups will be defined using natural bins (if they are apparent) or percentiles. Then, within each stratum, an unmatched approach will be used to calculate the win ratio. The overall win ratio will be estimated using the Mantel–Haenszel-type weight and reported with a 97.5% confidence interval. The method for adjustment will be described in detail in the SAP and finalized prior to data lock. Sensitivity analysis will be conducted with the same analysis in the HCUmITT population to assess the robustness of the effect found in the primary analysis.

##### Secondary outcomes

Analysis of each element of the composite healthcare utilization metric will include separate logistic models for ICU care, unplanned hospitalization, additional unplanned procedure, ED visit, and unplanned clinic visit or testing endpoints. A Poisson model will be used to assess the number of ambulatory patient–provider interactions. The randomized cohort models will include the treatment group and stratification factor (stone location (kidney or ureter)) as independent variables. The observational cohort will include a treatment group and adjust for patient/surgical covariates (age, sex, body mass index (BMI), Charlson comorbidity index, stone location (kidney or ureter), and stone size) as independent variables. These analyses will be performed with the ITT population with a sensitivity analysis using the HCUmITT.

Additional HRQOL endpoints, including PROMIS® Pain Interference scores at 4–6 weeks change from preoperative scores, PROMIS® Pain Intensity *t*-score changes, and NIH LURN SI-10 urinary symptom score changes will be analyzed using ANOVA models as described for the pain interference primary endpoint analysis based upon cohort (RCT or observational). Similarly, ANOVA models will be used to assess ICIQ-S treatment satisfaction scores at postoperative days 7–10 and 4–6 weeks separately. These models will be similar to the primary analysis models based upon cohort for the pain interference primary endpoint except a preoperative score does not apply as satisfaction is only measured after the surgery. Alternatively, if the trajectory of change for the outcome is found to be linear, then a linear mixed model will be used to compare scores at 7 days and 4–6 weeks between treatment arms. The mixed models will add the independent variables of time and the interaction of arm and time, a random effect in the model for patient and an autoregressive covariance matrix. Each analysis will report means with 95% CI at each time for each group, with a model-based type-3 *F*-test comparing treatment groups.

Number of days off work will be described using medians with interquartile ranges by treatment arm in each cohort. Linear models like the treatment satisfaction score models will be used based upon cohort. If the assumptions of the linear model are violated, Poisson models will be used.

### Interim analyses {21b}

An interim analysis after 396 patients are accrued is planned for confirmation of assumptions and re-estimation of sample size and power. Measures to be assessed include the proportion of patients who agree to join the randomization trial arm, the overall standard deviation of the primary endpoint, pain interference at days 7–10 change from pre-surgery, the proportion stented in the observational arm, and the intraclass correlation (ICC) in the observational arm. The sample size and power of each trial cohort will be reassessed based upon the interim analysis estimates compared to the assumed estimates. The results will be shared with the trial team and discussed with the funder.

### Methods for additional analyses (e.g., subgroup analyses) {20b}

Planned sub-group analyses will include an analysis by stone location for the primary and secondary endpoints. The methods will be similar to the analysis plan for each endpoint. A fixed effect for the statistical interaction of stone location and treatment arm will be used in the statistical models to measure a signal for a difference in outcome by stone location. A separate analysis for each stone location will be performed for the health care utilization Win Ratio outcome using analysis methods described for each cohort in the primary analysis plan.

### Methods in analysis to handle protocol non-adherence and any statistical methods to handle missing data {20c}

The populations for analysis relating to protocol non-adherence will be defined as the following:

- Intent-to-treat (ITT): all patients consented who undergo ureteroscopy, complete a baseline PROMIS® Pain Interference questionnaire and fit the definition of an eligible ureteroscopy at the end of the procedure.
- Pain Modified Intent-to-Treat (PmITT): all patients consented who undergo ureteroscopy, complete a pre-surgery and days 7–10 PROMIS® Pain Interference questionnaire and fit the definition of an eligible ureteroscopy at the end of the procedure.
- Healthcare Utilization Modified Intent-to-Treat (HCUmITT): all patients consented who undergo ureteroscopy, complete a baseline PROMIS® Pain Interference questionnaire, fit the definition of an eligible ureteroscopy at the end of the procedure, and have complete healthcare utilization data collection for 30 days after the procedure.

For the planned ITT analyses, multiple imputation will be used to address missing data. The details for multiple imputation will be described fully in the statistical analysis plan.

### Plans to give access to the full protocol, participant-level data and statistical code {31c}

The full study protocol is available to the public at the trial’s public-facing web page: https://www.stentornot.com/study-documents. De-identified patient-level data from the trial will be archived in the PCORI Repository and reasonable requests for secondary use will be considered through standard procedures noted on the PCORI website: https://www.pcori.org/research/about-our-research/data-sharing-maximizing-utility-pcori-funded-data. Statistical code may be shared upon reasonable written request to the trial’s principal investigator.

## Oversight and monitoring

### Composition of the coordinating center and trial steering committee {5d}

SOUL is a patient-centered clinical trial. Its advisory structure reflects this priority through robust involvement of both expert and public/patient partners in every aspect of the trial’s design and conduct. Two groups will serve in ongoing advisory roles to help guide the successful implementation and performance of the trial: an investigative team of physicians, researchers, and other coordinating center staff and a stakeholder engagement group that includes patient partners and advocates in kidney stone disease. The principal investigator has ultimate responsibility for overseeing both the investigative team and the stakeholder engagement group.

The investigative team consists of the principal investigator and co-investigators with expertise in both content (ureteroscopic treatment of kidney stone disease, patient-reported outcomes) and process (pragmatic clinical trial design and management, qualitative assessments of patient and physician preferences) relevant to the study, statistical analysts, project managers, and trial coordinators. They will meet at least monthly for the duration of the trial to review progress, discuss any needed modifications or adaptations, and facilitate ongoing success. A core team of the PI and MUSIC staff conducting the staff will meet weekly to ensure study enrolment and milestones are met.

The Stakeholder Engagement Group is co-led by the principal investigator and one co-investigator (NC), with expertise in stakeholder engagement, and also includes patients with experience of ureteroscopy, patient advocacy organizations, a payor representative, and physicians with experience of executing large scale patient-centered clinical trials. The stakeholder engagement group will meet at least two times annually to discuss study progress, as well as any problems that have been encountered regarding recruitment, enrolment, or retention. As the trial nears completion, we will also discuss dissemination efforts and seek out guidance for assistance with dissemination to the broader community.

In addition to the advisory panels described above, we will also hold annual Full Project Team meetings to include all project investigators, staff, stakeholder engagement group representatives, performance site physician leads, and representatives from PCORI. The primary purpose of these meetings will be to maintain forward progress and address any issues that have arisen.

### Composition of the data monitoring committee, its role, and reporting structure {21a}

Safety oversight is under the direction of a Data and Safety Monitoring Board (DSMB) composed of individuals with the appropriate expertise, including experience of clinical trials for stone surgery patients, and appropriate biostatistics knowledge for clinical trials. Members of the DSMB are independent from the study conduct and free of conflict of interest. The DSMB will meet at least semi-annually to assess safety and efficacy data on each arm of the study. The DMSB will operate under the rules of an approved charter that was written and reviewed at the organizational meeting of the DSMB. The DSMB will provide its input to PCORI through regular reports.

### Adverse event reporting and harms {22}

The principal investigator and co-investigators are responsible for the detection, documentation, grading, and assignment of attribution and expectedness of events meeting the criteria and definition of an adverse event. Adverse events will be collected by review of the electronic health record, ongoing communication with patients and participating performance site clinicians, and a text message query to patients at postoperative day 30. The DSMB will regularly review data on adverse events as a part of their ongoing safety reviews. Adverse events meeting standard criteria for classification as “serious adverse events” will be reported to the governing IRB.

For the purposes of this trial, an adverse event is considered any untoward medical occurrence in a patient receiving study treatment that has or potentially has a causal relationship with this treatment. Any untoward medical occurrences that are unrelated to the study intervention will not be considered adverse events. Symptoms of kidney stone disease and expected side effects from ureteroscopy lithotripsy and stent placement are not considered adverse events for this study.

### Frequency and plans for auditing trial conduct {23}

Each clinical performance site will perform internal quality management of study conduct, data collection, documentation, and completion. Quality control procedures will be implemented beginning with the data entry system and data checks that will be run on the database. Any missing data or data anomalies will be communicated to the performance site for clarification/resolution.

The institutional review board will verify that the clinical trial is conducted, and data are generated, documented, and reported in compliance with the protocol and applicable regulatory requirements.

The performance site will provide direct access to all trial-related sites, source data/documents, and reports for the purpose of monitoring and auditing by the MUSIC coordinating center and inspection by local and regulatory authorities.

### Plans for communicating important protocol amendments to relevant parties (e.g., trial participants, ethical committees) {25}

Any amendment to the study protocol and/or informed consent will require review and approval by the IRB before the changes are implemented to the study. Any such amendments will also be provided to PCORI for review and feedback prior to implementation.

### Dissemination plans {31a}

This study will comply with all applicable PCORI policies, including but not limited to the PCORI Policy for Data Management and Data Sharing and the PCORI Process for Peer Review of Primary Research and Public Release of Research Findings. Trial results will be published in peer-reviewed scientific journals, and a layperson summary of key findings will be made available to the public.

## Discussion

The SOUL trial addresses shortcomings of prior studies by (1) assessing PROs (HRQOL, symptoms, and treatment satisfaction), (2) assessing objective standardized healthcare outcomes, (3) using an integrated trial design in a real-world setting, (4) using RAND Appropriateness Criteria for comparative effectiveness studies, and (5) improving the evidence base on stent decision-making by using a mixed methods approach. Furthermore, the study design was based on a multi-year process whereby we used a participatory action approach from patient partners to ensure we assessed patient-centered outcomes and identified the issue of stenting as a significant problem when undergoing kidney stone surgery.

Furthermore, SOUL leverages the existing interpersonal and professional networks within a surgical quality improvement collaborative (MUSIC) to strengthen and support the trial’s success. All centers used a standardized pain protocol pathway which is a strength of this study. Tri-annual MUSIC consortium-wide meetings are held to discuss data, review risk-adjusted measures of processes of care and patient outcomes, and identify strategies and best practices for quality improvement. We will use this forum to engage with urologists about the SOUL trial as well as regular working group meetings that MUSIC conducts on a tri-annual basis.

As a multicenter prospective trial, the success of the SOUL study will depend on sustained engagement and participation by investigators and site champions across a broad spectrum of urologic practices throughout Michigan and beyond. This will be achieved in part by utilizing the existing sense of community and regular cadence of both in-person and virtual meetings between members of MUSIC. Investigators and other key personnel from multiple trial performance sites can be refreshed about key aspects of the study, provided with progress updates, and recognized for their contributions at collaborative-organized functions. Similarly, issues that may arise at individual performance sites can be efficiently resolved and solutions quickly disseminated to the broader group through existing networks.

Limitations of the SOUL clinic trial include that it is not blinded, and that the type of stent, duration of stenting, and removal method are left at the discretion of the enrolling surgeon. This pragmatism allows variation which may have an impact on outcomes. However, the pragmatic nature of the study is also a strength as it will allow a greater chance of patient enrollment and participation. The variety of centers in the trial provides a diverse multi-center population. Finally, the patient input on trial design and conduct, with regular patient feedback and engagement, is a major strength.

In summary, the SOUL study is innovative and can be insightful for the scientific community because it provides a real-world assessment of patient outcomes from diverse community and academic centers. The combined randomized and observational cohort design captures the outcomes of patients who decline randomization and was developed in conjunction with patient partners. It offers a comprehensive evaluation of PROs for one of the most common procedures in urology, developed with input from patient partners. It assesses 30-day healthcare utilization using a novel hierarchical rank score (Win Ratio). It uses a web-based intraoperative randomization system that allows randomization to occur just before completion of the surgical procedure. It has a qualitative component: interviews and surveys of both surgeons and patients to understand decisions and preferences around stenting. During peer review, this proposed clinical trial was found to have the potential to be paradigm-shifting as the patient’s voice was being heard and incorporated. Finally, it was noted that successful completion of this scientifically rigorous study will likely positively impact the field of urology and help surgeons and patients make patient-driven informed health care decisions.

## Trial status

Recruitment commenced on 1 June, 2023. The current protocol is Version 1.0 of 1 June, 2023. As of May 30, 2024, we have enrolled 162 total patients (78 in the randomized cohort and 84 in the observational cohort). Enrollment is estimated to complete on June 2025.

## Data Availability

The full data package from the study will be prepared in accordance with PCORI Methodology Standards for Data Integrity and Rigorous Analysis and will be deposited in a PCORI-designated repository and maintained for at least 7 years following study completion. The datasets used and/or analyzed during the current study will be made available from the corresponding author upon reasonable request.

## Abbreviations

EMA: Ecological momentary assessment
HRQOL: Health-related quality of life
ICIQ-S: International Consultation on Incontinence Questionnaire Satisfaction
ICU: Intensive care unit
IRB: Institutional review board
LURN SI-10: Symptoms of Lower Urinary Tract Dysfunction Research Network 10-item Symptom Index
MUSIC: Michigan Urological Surgery Improvement Collaborative
PCORI: Patient-Centered Outcomes Research Institute
PRO: Patient-reported outcome
PROMIS: Patient-Reported Outcomes Measurement Information System
REDCap: Research Electronic Data Capture system
RCT: Randomized clinical trial
SOUL: Stent Omission after Ureteroscopy and Lithotripsy
